# An explainable prognostic prediction panel for sepsis based on serum amino acid profiles

**DOI:** 10.3389/fimmu.2026.1757924

**Published:** 2026-06-01

**Authors:** Yue Liu, Long Zhao, Mingyue Sun, Jingyao Zhang, Chong Gu, Nanbin Hu, Shuangshuang Gu, Yan Shi

**Affiliations:** 1 Department of Emergency and Critical Care Medicine, Huai’an Second People’s Hospital, Huai’an, China; 2 The Affiliated Lianshui County People’s Hospital of Kangda College of Nanjing Medical University, Huai’an, China; 3 Department of Emergency, Nanjing Drum Tower Hospital, The Affiliated Hospital of Nanjing University Medical School, Nanjing, China

**Keywords:** machine learning, prognostic prediction, sepsis, serum amino acids, SHAP

## Abstract

**Background:**

Sepsis is a life-threatening syndrome requiring aggressive management, and novel noninvasive biomarkers to enable high-confidence risk stratification of sepsis and to predict sepsis-related outcomes are urgently needed.

**Methods:**

Quantitative (liquid chromatography) mass spectrometry was used to compare the abundance of serum amino acids between patients with sepsis and healthy controls (HC) to characterize alterations in amino acid profiles associated with sepsis. In addition, multiple machine learning (ML) methods were applied to construct a prognostic prediction model for patients with sepsis. The predictive performance was assessed, and the feature contributions were screened, followed by the development of an explainable prognostic prediction panel for sepsis.

**Results:**

Sixty participants in the HC group and 172 patients in the sepsis group (82 patients with septic shock) were included in this study. A discernible difference in the amino acid profiles between the HC and sepsis groups was detected, and the abundance of amino acids differed significantly among the HC, septic shock, and non-septic shock groups, indicating that amino acids could differentiate patients with sepsis from the HC group with good diagnostic performance. Then, 172 patients in the sepsis group were assigned to training and validation sets, and 130 patients with sepsis in the external test set were included. Five ML models (deephit, piecewise constant hazard, probability mass function, resource selection function, and extreme gradient boosting) were subsequently used, and the deephit model was selected according to the greater area under the curve and clinical benefits in the training, validation, and test sets. In the process of reducing features on the basis of feature importance rankings, the Deephit model, which is based on five features, had the best ability to predict survival probability, and an optimized Deephit model, which involves screening five features, namely, glutamine, glycine, lysine, pyroglutamic acid and proline, was successfully developed to predict the prognostic risk probability for patients with sepsis.

**Conclusions:**

Collectively, our findings demonstrated that alterations in serum amino acid profiles distinguish patients with sepsis from HCs and are strongly associated with clinical outcomes, supporting their utility as candidate features for prognostic prediction models in sepsis.

## 1 Introduction

Sepsis is a dangerous medical emergency that happens when the body’s defense system overreacts to an infection, damaging its own organs (1). Globally, tens of millions of people suffer from sepsis, a condition characterized by complexity, variability, and a notably high mortality rate (2). Septic shock, the severe stage of sepsis, has a mortality rate of up to 40% (3). Despite substantial advancements in treatments, such as fluid resuscitation and anti-infective strategies, there is a growing consensus that sepsis is a life-threatening syndrome requiring aggressive management (4). Early detection, assessment of sepsis severity, and appropriate treatment can significantly decrease sepsis-related mortality. Currently, multiple clinical scoring systems, including the Sequential Organ Failure Assessment (SOFA), Quick SOFA (qSOFA), and Acute Physiology and Chronic Health Assessment (APACHE-II), have emerged as optimal for short-term sepsis prognosis (5). Furthermore, biochemical indicators such as C-reactive protein (CRP) and procalcitonin (PCT) have been identified as prognostic markers for patients with septic shock (6). However, most of these indicators are susceptible to interference from other diseases and clinician expertise and lack utility (6). Consequently, there is an unmet need for the identification of novel noninvasive biomarkers to enable high-confidence risk stratification of sepsis and to predict sepsis-related outcomes.

In patients with sepsis, energy metabolism is severely disrupted, primarily characterized by protein degradation, gluconeogenesis, and increased fat mobilization, which are likely related to abnormal inflammatory mediator production and hormone secretion (7, 8). Amino acid metabolism is crucial for protein synthesis, maintaining normal metabolic functions, enhancing immunity, and participating in inflammatory processes (9, 10). In previous studies, significant alterations in serum amino acid metabolism pathways between patients with sepsis and those without sepsis were revealed using non-targeted metabolomics, with serum amino acids emerging as potential biomarkers of sepsis (11). However, abnormal energy metabolism in sepsis can lead to persistent inflammation and organ dysfunction, thereby affecting patient prognosis (12). Notably, protein metabolic degradation plays a crucial role in preserving immune function, influencing patient body mass index, and substantially affecting clinical outcomes in patients with sepsis (13). These findings indicate a plausible link between sepsis prognosis and amino acid metabolism; however, research on the correlation between amino acid metabolism and the prognosis of patients with sepsis, as well as a prognostic prediction model based on serum amino acid profiles for patients with sepsis, is lacking.

**Figure 1:**
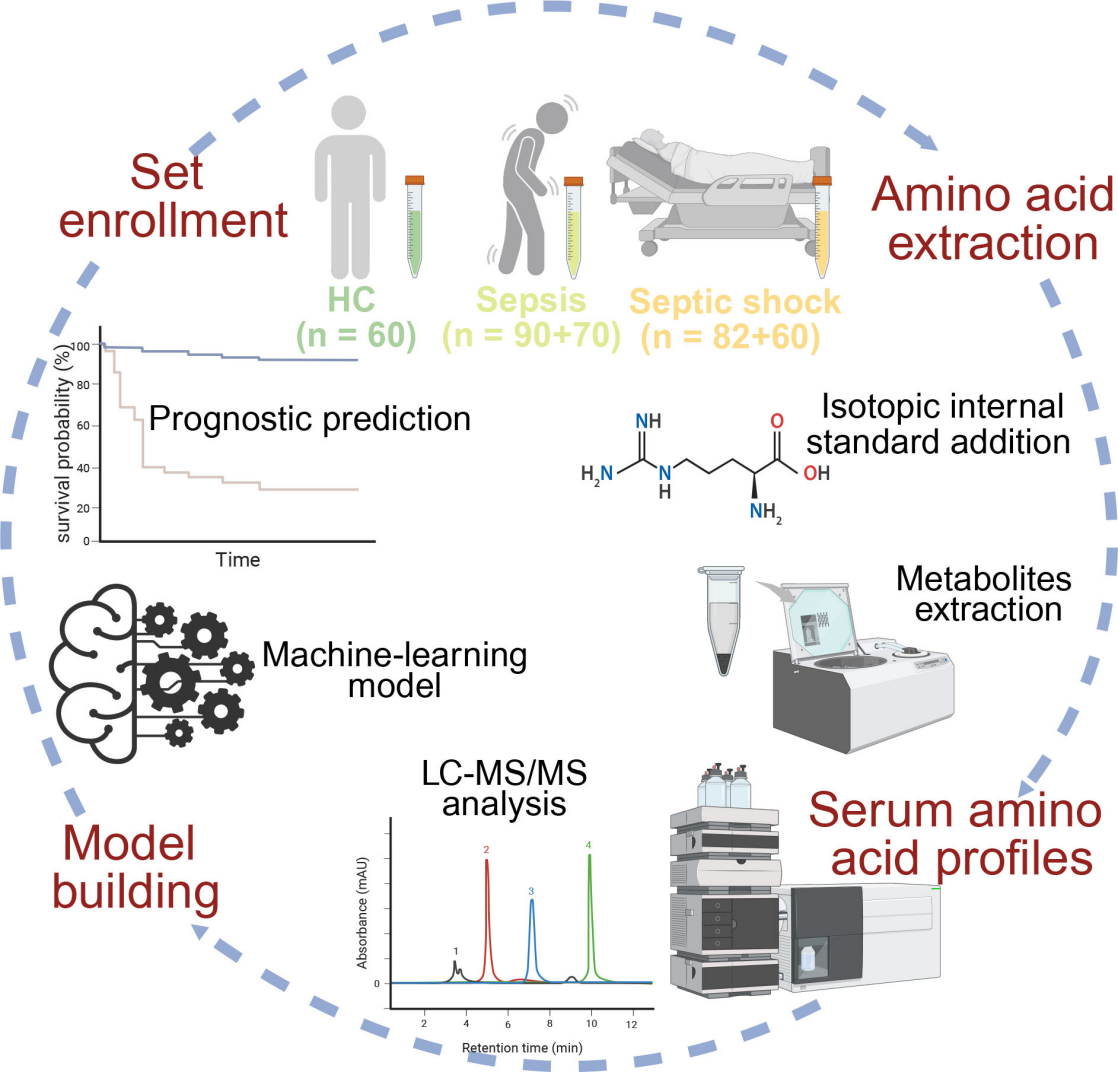
Study design of the analyzed set and experimental workflow identification and validation of an explainable prognostic prediction panel for sepsis on the basis of amino acid profiles.

Therefore, in this study, we aimed to use liquid chromatography–tandem mass spectrometry (LC–MS/MS) to quantitatively analyze serum amino acid levels in patients with sepsis and to explore amino acid alterations associated with disease prognosis. Furthermore, we sought to develop an interpretable machine learning (ML)-based prognostic model to identify candidate biomarkers for the outcome prediction and monitoring of sepsis (**Figure 1**). Overall, this approach may offer new insights into the amino acid basis of sepsis and contribute to more precise and individualized prognostic assessments in clinical practice.

## 2 Materials and methods

### 2.1 Chemicals and reagents

A total of 35 amino acid metabolites, including 3-methyl-L-histidine (3Mehis), alanine (ALA), arginine (ARG), asparagine (ASN), aspartic acid (ASP), cystine (CYS), gamma-aminobutyric acid (GABA), glutamic acid (GLU), glutamine (GLN), glycine (GLY), glycocyamine (GAA), histidine (HIS), homocitrulline (HCit), hydroxyproline (HYP), isoleucine (ILE), kynurenine (KYN), leucine (LEU), lysine (LYS), methionine (MET), N,N-dimethylglycine (NNDMG), N6-acetyl-L-lysine (N6AL), N-acetylcysteine (NAC), ornithine (ORN), phenylalanine (PHE), pipecolic acid (PIP), proline (PRO), pyroglutamic acid (PGA), S-adenosyl-L-homocysteine (SAH), serine (SER), S-methyl-L-cysteine (SMC), threonine (THR), tryptophan (TRP), tyrosine (TYR), 5-aminovaleric acid (5AA), and 5-hydroxy-L-tryptophan (5HTRP) standards, were purchased from Shanghai Aladdin Reagent Company. The isotopic internal standard 13C5-L-glutamic acid was obtained from Cambridge Isotope Laboratories, Inc., while MS-grade methanol, acetonitrile, and formic acid were obtained from Sigma, USA.

### 2.2 Study participants

This retrospective study included patients diagnosed with sepsis and septic shock at the Emergency Intensive Care Center of Huai’an Second People’s Hospital and Nanjing Drum Tower Hospital between December 1, 2018, and May 1, 2023, at The Affiliated Lianshui County People’s Hospital of Kangda College of Nanjing Medical University between March 1, 2022, and July 1, 2025. The sepsis diagnosis was consistent with the criteria outlined in Sepsis 3.0, wherein patients with a SOFA score of 2 or higher due to infection were included (14). Septic shock was defined as persistent hypotension, requiring vasoactive drugs to maintain a mean arterial pressure (MAP) ≥65 mmHg (1 mmHg=0.133 kPa) and a serum lactate concentration greater than 2 mmol/L (>18 mg/dL) in the absence of hypovolemia (14). The exclusion criteria were as follows: (1) pregnancy status; (2) age <18 years or >80 years; (3) receiving antibiotic treatment within 3 months before inclusion; (4) combined with immunosuppressive diseases (malignant tumors, immunosuppressive treatment, organic disease or drug-induced reduction in immune cells); (5) combined with endocrine metabolic diseases (such as hyperthyroidism, hypothyroidism, pheochromocytoma, primary aldosteronism, etc.); (6) rejection or patients who did not cooperate with treatment; and (7) missing clinical data. Healthy volunteers matched at the physical examination center (age, sex, height and weight) were also included in the healthy control (HC) group. This study was conducted in accordance with the Declaration of Helsinki and approved by the Human Ethics Committee of Huai’an Second People’s Hospital, Nanjing Drum Tower Hospital and Lianshui County People’s Hospital. Written informed consent was obtained from all participants or their legal representatives prior to enrollment.

### 2.3 Collection of demographic and clinical characteristics

Blood samples were collected from the patients within 24 hours of the diagnosis of sepsis or septic shock (14). The demographic and clinical characteristics of the patients, including age, sex, medical history, vital signs (mean arterial pressure, heart rate, respiratory rate, and temperature), and routine laboratory results (creatinine level, total bilirubin level, platelet count, CRP level, PCT level, lactic acid level, white blood cell count [WBC], and SOFA score), were recorded. The 60-day and 30-day outcomes of patients were assessed, with follow-up endpoints determined according to patient outcomes.

### 2.4 Patient grouping

Patients with sepsis were categorized into septic shock and non-septic shock groups based on Sepsis 3.0 diagnostic criteria. In accordance with the inclusion and exclusion criteria, a total of 172 patients with sepsis (82 patients with septic shock) were initially enrolled. These patients were randomly divided into training and validation sets for model development. In addition, an independent external test cohort comprising 130 patients with sepsis was collected from The Affiliated Lianshui County People’s Hospital of Kangda College of Nanjing Medical University. Furthermore, 60 healthy volunteers who were matched at the physical examination center and assigned to the HC group were enrolled for comparison (**Supplementary Figure 1**).

### 2.5 Quantitative analysis of serum amino acid profiles

Fresh blood samples were centrifuged at 3000 rpm for 15 min, and serum samples were transferred to Eppendorf tubes, which were numbered and stored in a −80 °C freezer. For the HC group, 2 mL of venous blood was collected from the blood drawn during physical examination. Blood samples were processed in the same manner as those obtained from patients with sepsis. An Agilent 1290 liquid chromatography system with an Agilent G6460 triple quadrupole mass spectrometer under optimized conditions in the positive ion mode of electrospray ionization (ESI) was used for amino acid profiling.

A targeted quantitative method based on ultra-performance liquid chromatography–tandem mass spectrometry (UPLC–MS/MS) was established to determine 35 amino acids in serum samples. The mass spectrometric parameters for each amino acid and the internal standard were first optimized, including the selection of precursor ions (Q1) and product ions (Q3), as well as the optimization of the declustering potential and collision energy to achieve the highest sensitivity and specificity. Multiple reaction monitoring (MRM) mode was subsequently developed for the simultaneous detection and quantification of all the analytes. Chromatographic separation was performed using a Poroshell 120 HILIC column (2.1 × 100 mm, 2.7 µm). The mobile phase consisted of (A) 20 mM ammonium formate in acetonitrile (water:acetonitrile = 1:9, v/v) and (B) 20 mM ammonium formate in water. Gradient elution was applied as follows: 100% A at 0 min, decreased to 70% A at 11.5 min, maintained at 70% A until 12 min, and then returned to 100% A at 15 min. The flow rate was set at 0.3 mL/min, the column temperature was maintained at 25 °C, and the injection volume was 5 µL. Mass spectrometric detection was carried out in positive electrospray ionization (ESI) mode. Multiple scan segments were employed with different collision energy (CE) settings to optimize fragmentation and enhance the signal intensity for each analyte. The MRM transitions of each amino acid and the internal standard were established to ensure accurate quantification (**Supplementary Table 1**). The optimized MS parameters were as follows: drying gas temperature, 330 °C; drying gas flow, 13.0 L/min; nebulizer pressure, 35 psi; sheath gas temperature, 390 °C; sheath gas flow, 12.0 L/min; and capillary voltage, 1,500 V. Quantification was performed using an internal standard method. A mixed standard solution containing all target amino acids and 13C5-L-glutamic acid as the internal standard was used to construct calibration curves. Linear regression analysis was performed by plotting the peak area ratio of each amino acid to the internal standard (y-axis) against the corresponding analyte concentration (x-axis). The linearity of the calibration curves was evaluated, and the lower limit of detection (LOD) and lower limit of quantification (LLOQ) were determined on the basis of signal-to-noise ratios. Method validation was conducted in accordance with standard bioanalytical guidelines. Quality control samples at four concentration levels-LLOQ, low (LQC), medium (MQC), and high (HQC)-were analyzed in five replicates over three consecutive days. Accuracy, intra-day precision, and inter-day precision were calculated to evaluate the reliability and reproducibility of the method. Data acquisition and processing, including extraction of retention time and peak area, were performed using Agilent MassHunter Qualitative Analysis software (version B.06.00).

### 2.6 Statistical methods

Normally distributed data are presented as the mean ± standard deviation (x ± s) and were compared using independent t tests and analysis of variance. Nonnormally distributed data were compared using the Mann–Whitney U test. Count data are expressed as percentages (%), and comparisons between groups were performed using the chi-square test. General statistical data were analyzed using SPSS software (version 26.0).

Differences in the abundance of amino acids between the HC group and sepsis group were analyzed using principal component analysis (PCA), orthogonal least squares discriminant analysis (OPLS-DA), and partial least squares discriminant analysis (PLS-DA) multivariate models. PCA serves as an unsupervised dimension reduction analysis (15), whereas OPLS-DA and PLS-DA are supervised multivariate statistical methods (16). These methods categorize datasets, filter out extraneous information to focus on the most pertinent factors within the principal components, and facilitate comparisons between multiple groups.

ML models, including Deephit (17), piecewise constant hazard (PCHazard) (18), probability mass function (PMF) (19), resource selection function (RSF) (20), and extreme gradient boosting (Xgboost) (21), were used to construct a 60-day prognostic prediction panel for patients with sepsis. Hyperparameter optimization based on a random search was performed, and K-fold cross validation was used to assess the generalization performance. Information about the packages and parameters of the ML models is listed in **Supplementary Table 2**. Time-dependent receiver operating characteristic (timeROC) curves and decision curve analysis (DCA) were used to evaluate the predictive ability and clinical application value of the models (22, 23). The sequential forward selection (SFS) approach was applied to iteratively identify the optimal feature subset (24), and SHapley Additive exPlanation (SHAP) was used to examine the contributions of amino acids and clinical characteristics in the Deephit model and was implemented to overcome the “black-box” issue (25). The nonparametric method of Delong was used to compare the difference between the area under the receiver operating characteristic curve and the concordance index (C-index) using MedCalc Version 22.0. Statistical significance was set at a P value < 0.05. Python (version 3.10.13) and R (version 4.0.3) software were used for the ML model construction and evaluation.

## 3 Results

### 3.1 Serum amino acid levels in patients with sepsis

**Figure 2:**
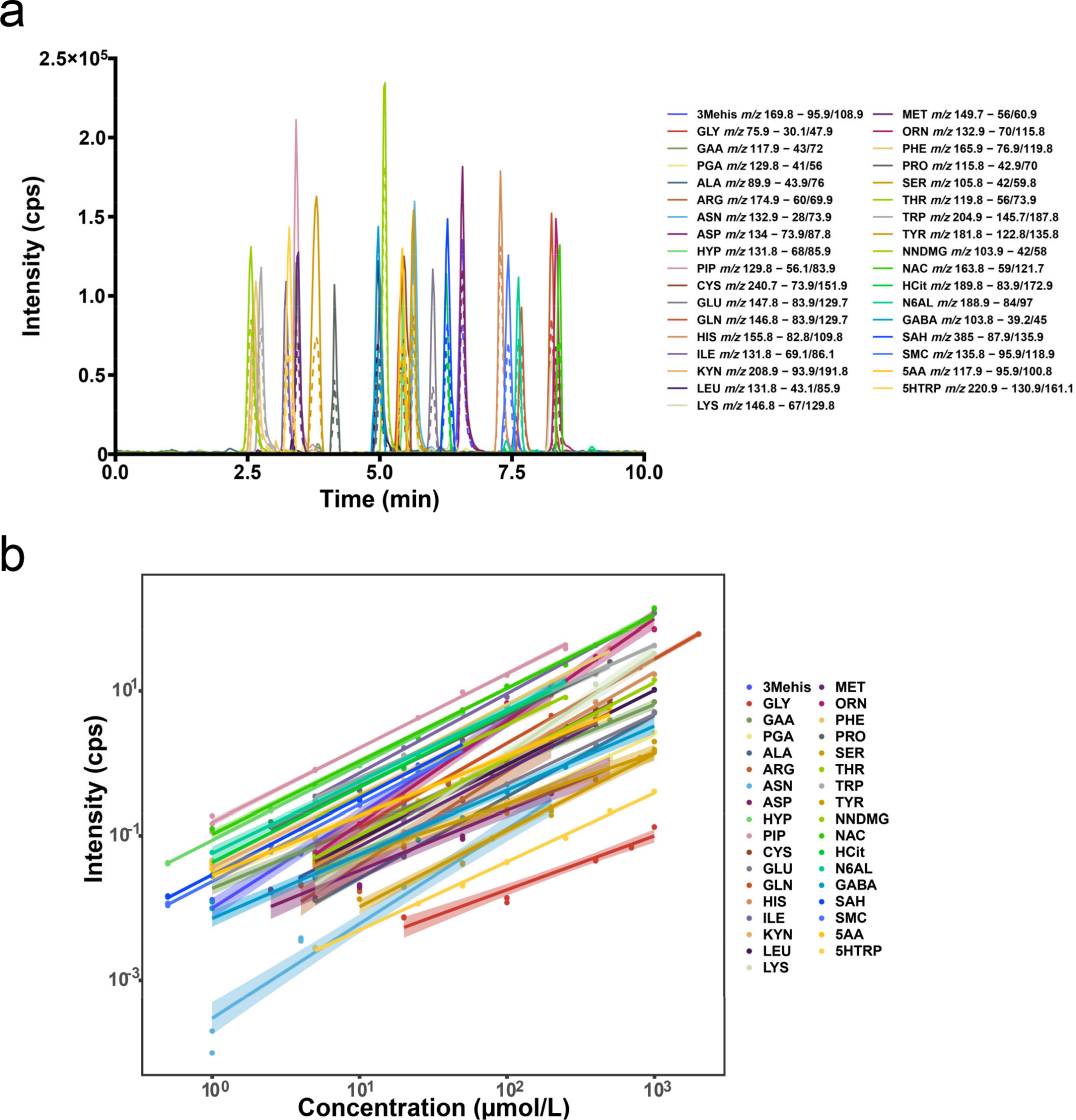
Development of amino acid profiles based on a liquid chromatography–tandem mass spectrometry (LC–MS/MS)-based quantitative MRM method. **(a)** A typical MRM chromatogram of target amino acids. **(b)** Calibration curves of target amino acids. The curves were constructed by plotting the mass spectrometry signal ratios of the amino acids and internal standards against the concentrations of the calibration standards.

A total of 172 patients with sepsis (82 patients with septic shock) with a mean age of 57 (45–68) years were included and randomly assigned to the training and validation cohorts, while an additional 130 patients with sepsis (82 patients with septic shock) with a mean age of 61 (49–74) years were enrolled as an independent external test cohort. In addition, 60 participants in the HC group with a mean age of 58 (47–71) years were enrolled in this study. No significant differences were observed in sex or age between the HC and sepsis groups. Univariate analysis revealed significant differences in temperature, heart rate, WBC count, PCT level, and SOFA score (P <0.05) between the sepsis group and the HC group, while the other indicators did not significantly differ (**Supplementary Table 3**). The MRM transitions of amino acids were successfully established for targeted quantification. Complete elution and effective separation of 35 amino acids were achieved within 0–10 min (**Figure 2a**). The calibration curves demonstrated excellent linearity, with all R² values exceeding 0.99 (**Figure 2b**, **Supplementary Table 4**). The abundance of serum amino acids in patients with sepsis was determined, and most serum amino acids could be quantified, except 5AA and 5HTRP, which were not detected in more than 50% of the samples; thus, no missing data were present for these variables in the final dataset used for model development. The abundance of the remaining 33 amino acids was used for subsequent analysis.

**Figure 3:**
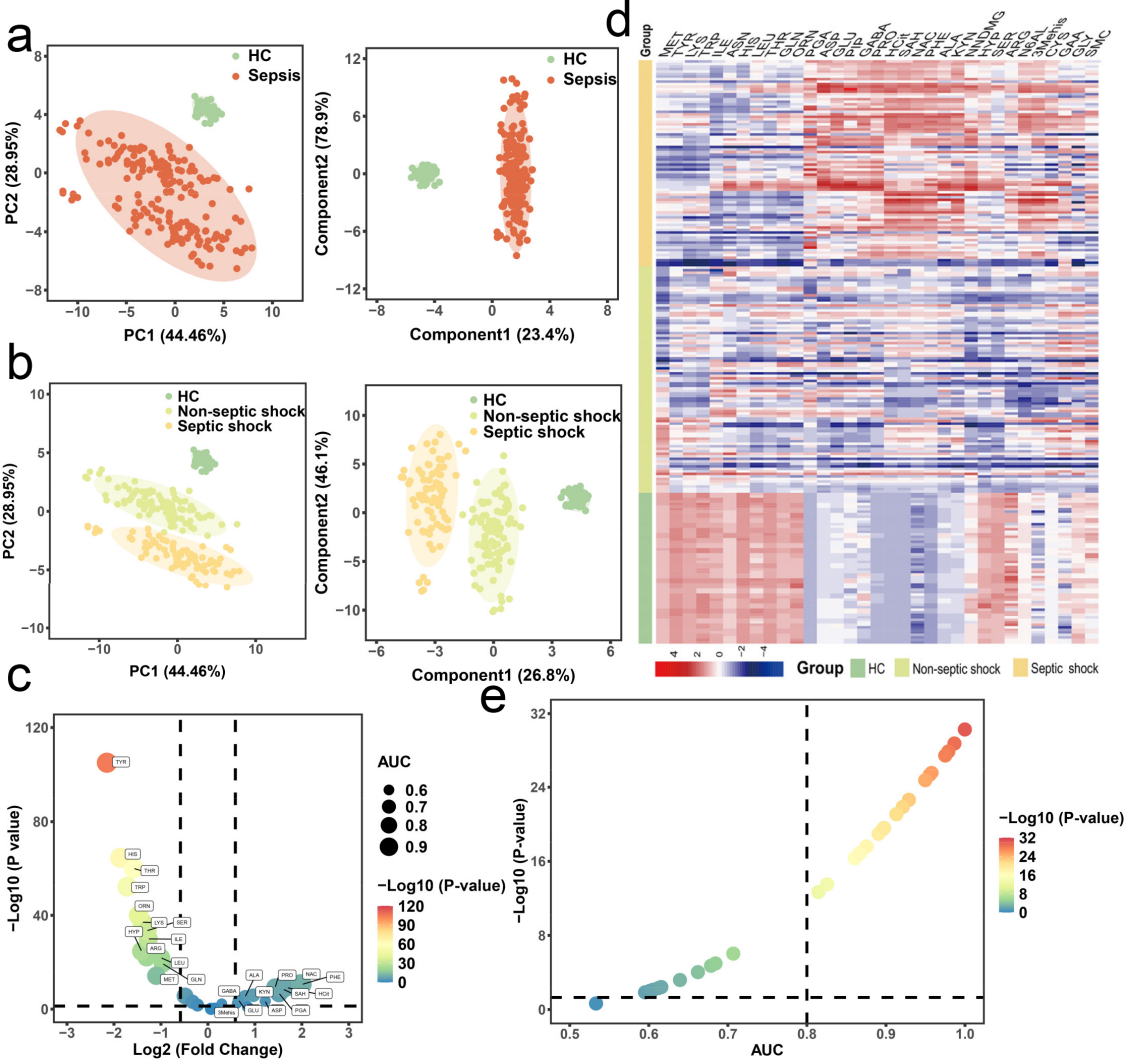
Abundance of amino acid profiles in patients with sepsis. **(a)** PCA, Principal component analysis (left) and OPLS-DA, orthogonal least squares discriminant analysis (right) plots of the HC, healthy control and sepsis groups. **(b)** PCA (left) and PLS-DA, partial least squares discriminant analysis (right) plots of the HC, non-septic shock, and septic shock groups. **(c)** Volcano plots of amino acid abundance between the HC and sepsis groups. **(d)** Heatmap of differentially expressed amino acids. **(e)** AUC, Area under the curve of the ROC, receiver operating characteristic curve and P-values of individual amino acids distinguishing the sepsis group from the HC group.

PCA and OPLS-DA revealed a discernible segregation trend in the amino acid profiles between the HC and sepsis (including septic shock) groups, while the prediction parameters of OPLS-DA were R2X = 0.843, R2Y = 0.970, and Q2 = 0.966 (**Figure 3a**). Furthermore, according to the definition criteria for Sepsis 3.0, all patients with sepsis were categorized into the septic shock and non-septic shock subgroups. Similarly, the PCA and PLS-DA results revealed that the abundance of amino acids differed significantly among the HCs, patients with septic shock, and patients with sepsis who did not develop septic shock, in which the PLS-DA parameters were R2X = 0.882, R2Y = 0.961, and Q2 = 0.942, respectively (**Figure 3b**), suggesting the robustness and predictive ability of serum amino acid profiles. Notably, intragroup homogeneity was evident within the sepsis and normal control groups as well as within the septic shock and non-septic shock groups (**Supplementary Figure 2**). We further calculated the fold change (FC) and P-value between the groups (HC *vs.* sepsis), which are presented in the form of a volcano plot (**Figure 3c**). │FC│> 1.5 and a P-value < 0.05 indicated significant alterations in abundance. Hierarchical clustering heatmap analysis based on Euclidean distance revealed correlations and expression trends in the HC, septic shock, and non-septic shock groups (**Figure 3d**), indicating overall differences in the serum amino acid profiles among the three groups. The area under the curve (AUC) and P-values of the ROC analyses for individual amino acids are shown in **Figure 3e**, indicating that serum amino acids exhibit a certain discriminatory ability between patients with sepsis and HCs. Consequently, these findings underscore the significant differences in the serum amino acid profiles between the HC and sepsis groups and between the sepsis subgroups.

### 3.2 Construction and validation of clinical prediction models based on the serum amino acid profiles and clinical characteristics

**Figure 4:**
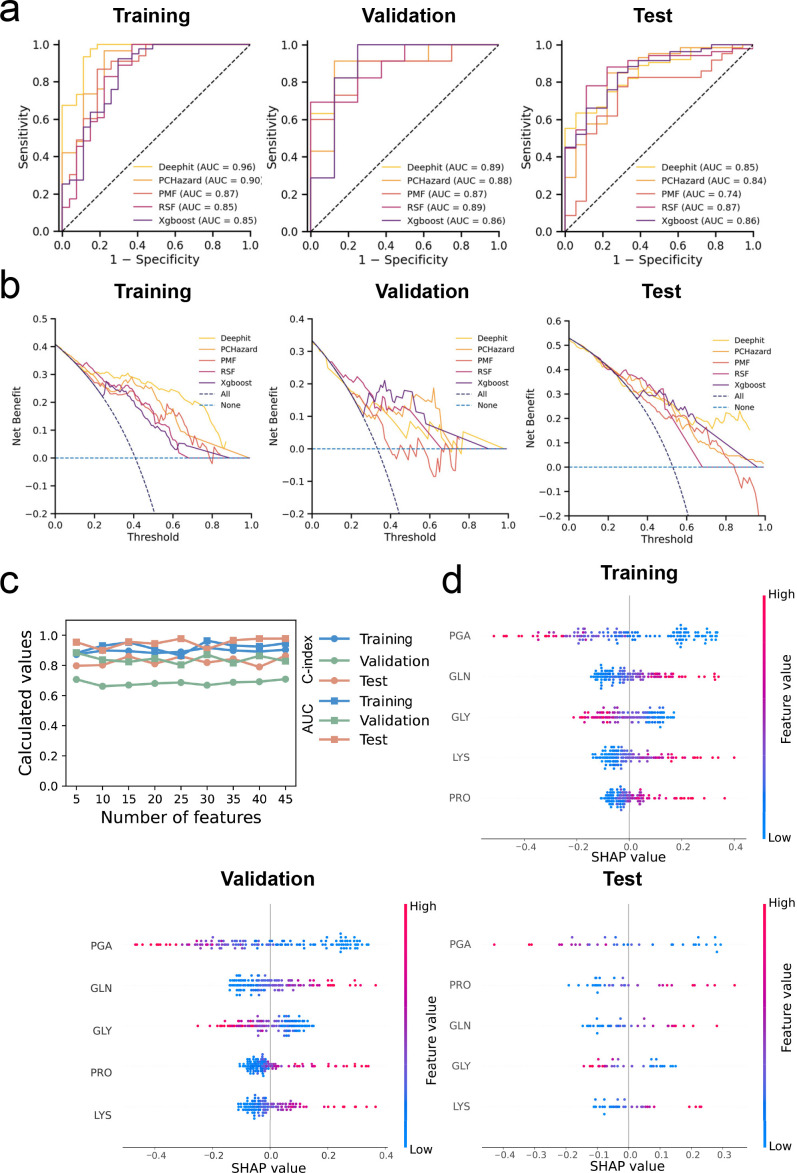
Construction and validation of clinical prediction models based on serum amino acid profiles and clinical characteristics. **(a)** timeROC, Time-dependent receiver operating characteristic analyses of the five ML models (Deephit, Pchazard, PMF, RSF, and XGgboost) were performed to construct a 60-day survival probability prediction model. **(b)** DCA, Decision curve analysis of the five ML models to evaluate the clinical efficacy and net benefits. **(c)** The Deephit model based on five features had the best ability to predict 60-day survival probability in the process of reducing features based on feature importance ranking. **(d)** SHAP values of the selected five selected features of the Deephit model.

Based on the aforementioned results, which highlight the pivotal role of serum amino acid profiles in sepsis diagnosis, we aimed to combine amino acid variability with 16 basic physical signs, which are shown in **Supplementary Table 2**, to formulate a prognostic prediction panel for individuals with sepsis. All patients with sepsis were randomly grouped into training and validation sets at a ratio of 8:2, and 130 patients with sepsis in the external test set were included. Five ML models (Deephit, Pchazard, PMF, RSF, and Xgboost) were subsequently used to construct a 60-day survival probability prediction model for patients with sepsis. The AUC values for the training set across the five models were 0.96, 0.90, 0.87, 0.85 and 0.85; those for the validation set were 0.89, 0.88, 0.87, 0.89 and 0.85; and those for the test set were 0.85, 0.84, 0.74, 0.87 and 0.86, respectively (**Figure 4a**). We subsequently used DCA to evaluate the clinical efficacy and net benefits of our model. Notably, all the ML models demonstrated favorable clinical utility and net benefits in the training set (**Figure 4b**). Given that the Deephit model exhibited greater AUC and clinical benefits in the training, validation and test sets, Deephit was selected for subsequent analysis. Notably, Deephit, being a “black-box” ML model, poses challenges in interpretation because of its opaque nature. To address this, we introduced the SHAP values to elucidate the feature contributions within the Deephit model. The SHAP values of the top 20 features of the Deephit model across the training, validation and test sets are shown in **Supplementary Figure 3**. To avoid potential information leakage, feature selection was performed exclusively on the training set. Based on SHAP-derived feature importance ranking, an SFS approach was applied to iteratively identify the optimal feature subset. As shown in **Figure 4c**, although increasing the number of features led to higher model complexity, the improvement in the AUC plateaued beyond five features. Therefore, the five-feature Deephit model (5F-Deephit) was selected as the final model (**Figure 4d**), balancing the predictive performance and model parsimony.

### 3.3 Application of the final Deephit model with five features

**Figure 5:**
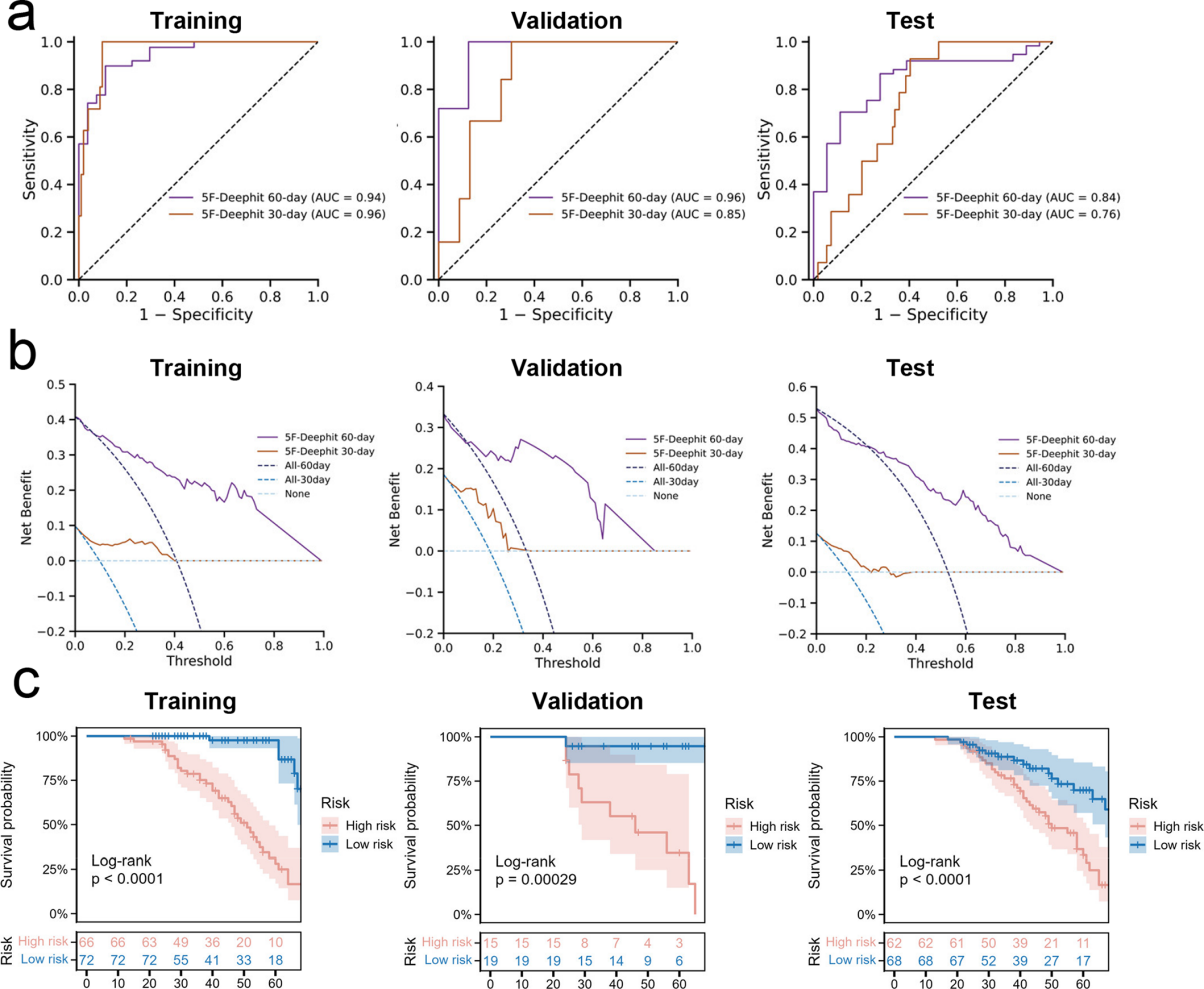
Application of the final Deephit model with five features. **(a)** timeROC, Time-dependent receiver operating characteristic analyses of the Deephit model with five features (5F-Deephit model) to construct a 60-day and 30-day survival probability prediction model. **(b)** DCA, Decision curve analysis of the 5F-Deephit model. Significant discrimination between the 60-day **(c)** survival probability of high-risk and low-risk patients.

In sepsis, 30-day mortality is widely used as a key short-term outcome, but mortality continues to increase beyond this period because of ongoing disease progression and complications. Therefore, assessing both 30-day and 60-day outcomes may provide complementary insights into early- and intermediate-term prognosis. We then used ROC curve analysis and DCA to evaluate the ability of the 5F-Deephit model to predict the 60-day and 30-day survival of patients with sepsis. The sensitivity and specificity were 0.96 and 0.71, respectively, with AUC values of 0.94 for predicting 60-day prognosis in the training set; 0.90 and 0.92, respectively, with AUC values of 0.96 for predicting 30-day prognosis in the training set; 0.93 and 0.96, respectively, with AUC values of 0.96 for predicting 60-day prognosis in the validation set; 0.83 and 0.92, respectively, with AUC values of 0.85 for predicting 30-day prognosis in the validation set; 0.94 and 1, respectively, with AUC values of 0.96 for predicting 60-day prognosis in the test set; and 0.89 and 0.81, respectively, with AUC values of 0.85 for predicting 30-day prognosis in the test set (**Figure 5a**). DCA also confirmed the clinical usefulness of the 5F-Deephit model in the training, validation and test sets (**Figure 5b**). These results indicated that the 5F-Deephit model has high predictive efficacy for the prognosis of patients with sepsis. The final explainable prognostic prediction model was implemented in a web application to facilitate clinical application, which can be accessed online at http://39.102.138.68:6003/ (**Supplementary Figure 4**). When the actual values of the five amino acids required by the model were entered, the application automatically predicted the prognostic risk probability for patients with sepsis. Additionally, we calculated and constructed the symptom formation risk probability for each patient in the training, validation and test sets based on the 5F-Deephit model. All patients with sepsis were categorized into low- and high-risk groups, according to an optimal cutoff threshold of 0.601 for a 60-day prognosis (**Supplementary Figure 5**) and 0.284 for a 30-day prognosis (**Supplementary Figure 6**). Patients with sepsis who died had greater 60-day and 30-day death risk probabilities in the training, validation and test sets than did patients who survived (**Supplementary Figure 7**). Most of the surviving patients were in the low-risk group in the training, validation and test sets (**Supplementary Figure 8**). Notably, Kaplan–Meier survival curves revealed significant discrimination between the 60-day (**Figure 5c**) survival probabilities of the high- and low-risk patients in the training, validation and test sets.

## 4 Discussion

To date, sepsis remains a prominent cause of hospitalization and mortality due to infections, with projections indicating concerns over the next two decades (26). In this study, we employed an LC–MS/MS-based quantitative MRM method to scrutinize the abundance of serum amino acids and developed an explainable prognostic prediction panel for patients with sepsis. Our findings revealed significant alterations in serum amino acid abundance in patients with sepsis compared with HCs, and these alterations were associated with patient prognosis.

Sepsis is accompanied by profound metabolic disturbances and nutritional deterioration (27). Most patients with sepsis exhibit a negative nitrogen balance, glycolipid dysregulation, and endocrine alterations, leading to a hypermetabolic state characterized by enhanced gluconeogenesis, increased fat mobilization, and impaired lipid utilization (28). In addition, abnormalities in lipid metabolism, including low-density lipoprotein clearance disorders and hypertriglyceridemia, further exacerbate metabolic imbalance (29). These amino acid changes reduce energy utilization efficiency and contribute to poor clinical outcomes (30). Consistent with these findings, previous studies have demonstrated that malnutrition is highly prevalent in sepsis patients and is closely associated with poor prognosis; for example, nutritional indices such as the prognostic nutritional index (PNI) and the Geriatric Nutritional Risk Index (GNRI) have been identified as independent predictors of mortality in patients with sepsis, with poor nutritional status significantly increasing mortality risk (31, 32). Moreover, a bidirectional interaction between sepsis and malnutrition has been proposed, in which systemic inflammation and hypercatabolism further aggravate nutritional depletion, thereby worsening disease progression and outcomes (31).

In this study, we constructed a prognostic model and revealed that pyroglutamic acid, glutamine, glycine, lysine and proline levels were closely associated with the prognosis of patients with sepsis. Pyroglutamic acid, also known as 5-oxoproline, is a natural amino acid and an intermediate of glutathione (GSH) metabolism, while elevated pyroglutamic acid levels may serve as a biomarker of GSH depletion and are correlated with oxidative stress (33). Previous research has shown that pyroglutamic acid is a metabolic marker of pathological lung damage in patients with tuberculosis and is closely associated with fulminant diabetes, suggesting its role in glucose metabolism (34). Additionally, pyroglutamic acid accumulation induces inflammation and impairs antioxidant defenses, potentially contributing to sepsis development, particularly in cases caused by *Pseudomonas aeruginosa* during burn infections (35). The abundance of glutamine often decreases during sepsis, and lower concentrations are generally associated with worse clinical outcomes (36). Some studies suggest that glutamine supplementation may influence immune function in sepsis patients, but its ability to improve prognosis remains uncertain (37). In animal models of sepsis, glycine has been shown to reduce inflammatory responses and organ damage, improve liver function, and prevent mortality, underscoring its protective effects against sepsis, endotoxins, and hemorrhagic shock (38). Lysine is an amino acid that is being studied for its potential to help treat sepsis-related complications, particularly sepsis-induced lung injury. Preclinical studies in animals have shown that lysine can reduce inflammation markers, oxidative stress, and organ damage associated with sepsis (39). Consistent with our findings, a previous study highlighted the relationship between significant variation in histidine biosynthesis and sepsis mortality (40). Proline, a nonessential cyclic amino acid, plays a crucial role in protein structure/function and the maintenance of cellular redox homeostasis (41). Its intracellular catabolism generates ATP and reactive oxygen species, which enhance immune function and promote energy metabolism (42). Recent studies have indicated that proline biosynthesis and catabolism are fundamental processes in diseases and influence a broad metabolic network through their roles in redox homeostasis (41). Proline metabolism is significantly associated with sepsis, as indicated by abnormally elevated plasma proline levels, which are correlated with disease severity and mortality and are positively correlated with the abundance of lactic acid (43).

Accurate prognostic prediction of sepsis is essential for guiding clinical decision-making and enabling personalized management strategies. Traditional approaches rely on clinical scoring systems such as SOFA and APACHE II, as well as biomarkers such as CRP and procalcitonin; however, these tools may be influenced by clinician experience, delayed physiological changes, and limited specificity. In contrast, our model is based on serum amino acid profiles, which reflect underlying metabolic and nutritional disturbances associated with sepsis pathophysiology and demonstrate robust predictive performance across multiple datasets, suggesting its value as a complementary tool to existing clinical predictors (44). In this study, multiple ML models, including Deephit, PCHazard, PMF, RSF, and XGBoost, each with distinct methodological characteristics and advantages, were evaluated for survival prediction. Traditional survival models such as PCHazard and PMF provide structured and interpretable hazard representations, which may be advantageous in settings where transparency is needed (19, 45). RSF extends random forest methods to survival data and can capture nonlinear relationships and complex feature interactions (20). XGBoost is a powerful ensemble learning method with strong performance in structured data, although it requires adaptation for time-to-event outcomes (21). To ensure a fair comparison, systematic hyperparameter optimization was performed for each model using a random search strategy combined with cross-validation within the training dataset, allowing all the models to be evaluated under their optimal configurations. Among these models, Deephit, a deep learning–based survival model, directly models the joint distribution of event occurrence and survival time, enabling flexible modeling of nonlinear relationships and temporal dependencies. In our study, Deephit consistently demonstrated superior predictive performance across the training, validation, and external test cohorts, suggesting its suitability for modeling the heterogeneous and time-dependent nature of sepsis. Importantly, this does not imply that other models are inferior; rather, each model has its own advantages depending on the data characteristics and clinical context. Despite the strong predictive performance of ML models, their interpretability remains a major challenge. To address the interpretability of the ML models, we applied SHAP, which enables intuitive visualization of feature contributions and facilitates the development of a simplified and interpretable prognostic model. Notably, although traditional clinical indicators such as the SOFA score and platelet count are well-established predictors of sepsis outcomes (46, 47), our findings suggest that amino acid profiles capture metabolic and nutritional alterations that are not fully reflected by conventional variables, which may explain their preferential selection in the final model. Taken together, the results of this study highlight two key advantages: the incorporation of amino acid profiling provides a metabolically informed perspective on sepsis heterogeneity, whereas the integration of ML enables automated feature selection and model optimization with maintained interpretability. These findings support the potential of combining amino acid profiles with ML to improve prognostic prediction and facilitate more precise, individualized clinical decision-making in sepsis patients.

Nonetheless, this study has several limitations. First, although ML technology requires “big data” to construct predictive models, there is no standard for calculating the sample size required for the development of ML-based predictive models (48). Although a preliminary analysis of the five selected amino acids was performed in a small cohort of non-septic critically ill patients (including multiple trauma and acute myocardial infarction; data not shown), further studies involving larger, well-characterized non-septic cohorts, as well as prospective multicenter designs, are warranted to better evaluate the generalizability and disease specificity of these findings. In addition, the heterogeneity of non-septic conditions may influence the interpretation of the results. Furthermore, the positive predictive value for the high-risk group, as defined by the cutoff derived from the abundance of the 5-amino acid–based integrated 5F-Deephit model, was relatively modest. Therefore, in clinical practice, high-risk patients should be identified in conjunction with other pathophysiological features and established biomarkers to avoid potential overestimation of risk and unnecessary interventions. In addition, our datasets were derived mainly from the abundance of amino acids, which was determined using LC–MS/MS-based quantitative MRM. Although mass spectrometry has been rapidly adopted in clinical settings, its availability remains limited in most primary healthcare settings (49).

In conclusion, we quantified serum amino acid levels in patients with sepsis and successfully developed an interpretable and explainable prognostic panel based on the abundance of serum amino acids. Based on the prognostic panel, early identification of high-risk patients enables prompt, targeted, and personalized treatments, improving sepsis clinical outcomes and reducing complications. This study revealed that alterations in serum amino acids play a pivotal role in the onset and prognosis of sepsis, suggesting the potential utility of these alterations in clinical practice and as therapeutic targets.

## Data Availability

The original contributions presented in the study are publicly available. This data can be found here: MetaboLights database, accession number MTBLS14567.
