# Supplementary material for: An explainable prognostic prediction panel for sepsis based on serum amino acid profiles
Source: Front Immunol. 2026 Jun 1;17:1757924. doi: 10.3389/fimmu.2026.1757924 (PMC13265505; doi:10.3389/fimmu.2026.1757924)
Supplement: Supplementary file 1 [file DataSheet1.docx]

An explainable prognostic prediction panel for sepsis based on serum amino acid profiles

Yue Liu^1^, Long Zhao^1^, Mingyue Sun^1^, Jingyao Zhang^1^, Chong Gu^1^, Nanbin Hu^3^, Shuangshuang Gu^2,*^, Yan Shi^1,*^

^1^Department of Emergency and Critical Care Medicine, Huai'an Second People's Hospital, Huai'an 223001, P.R. China

^2^Department of Emergency, Nanjing Drum Tower Hospital, The Affiliated Hospital of Nanjing University Medical School, Nanjing 210008, P.R. China

^3^The Affiliated Lianshui County People's Hospital of Kangda College of Nanjing Medical University, Huai'an 223001, P.R. China

*Corresponding author: Yan Shi, jfmsy@163.com; Shuangshuang Gu, [guss2926@njglyy.com](mailto:guss2926@njglyy.com)

**Table of Contents**

**Supplementary Table 1.** Optimization of MS parameters of target amino acids. DP delustering potential, CE collision of energy.

**Supplementary Table 2.** List of packages used for the analysis and optimal parameters for model.

**Supplementary Table 3.** Detailed Demographic and clinical characteristics of the enrolled participants.

**Supplementary Table 4.** Analytical performance parameters of the UPLC–MS/MS-based quantitative platform for amino acid analysis. The evaluated parameters include the linear range, limit of detection (LOD), lower limit of quantification (LLOQ), accuracy, and precision. Quality control (QC) samples at different concentration levels were used to assess intra- and inter-day precision, expressed as coefficient of variation (CV).

**Figure S1.** Flowchart of the inclusion and exclusion process of patients in this study.

**Figure S2.** Abundance of amino acid profiles in healthy control (HC) group, patients with sepsis and patients with septic shock.

**Figure S3.** SHapley Additive exPlanation (SHAP) values of the top 20 features of the Deephit model.

**Figure S4.** An online web application according to the final Deephit model with five features.

**Figure S5.** Construction of the symptom formation 60-day probability of death risk for each patient in the training and independent set based on the 5F-Deephit model.

**Figure S6.** Construction of the symptom formation 30-day probability of death risk for each patient in the training and independent set based on the 5F-Deephit model.

**Figure S7.** Statistical analysis of death risk probability between different clinical prognosis in both the training and test sets.

**Figure S8.** Statistical analysis of percentage of different clinical prognosis (60-day prognosis in the top row and 30-day prognosis in the bottom row) between high and low-risk group.


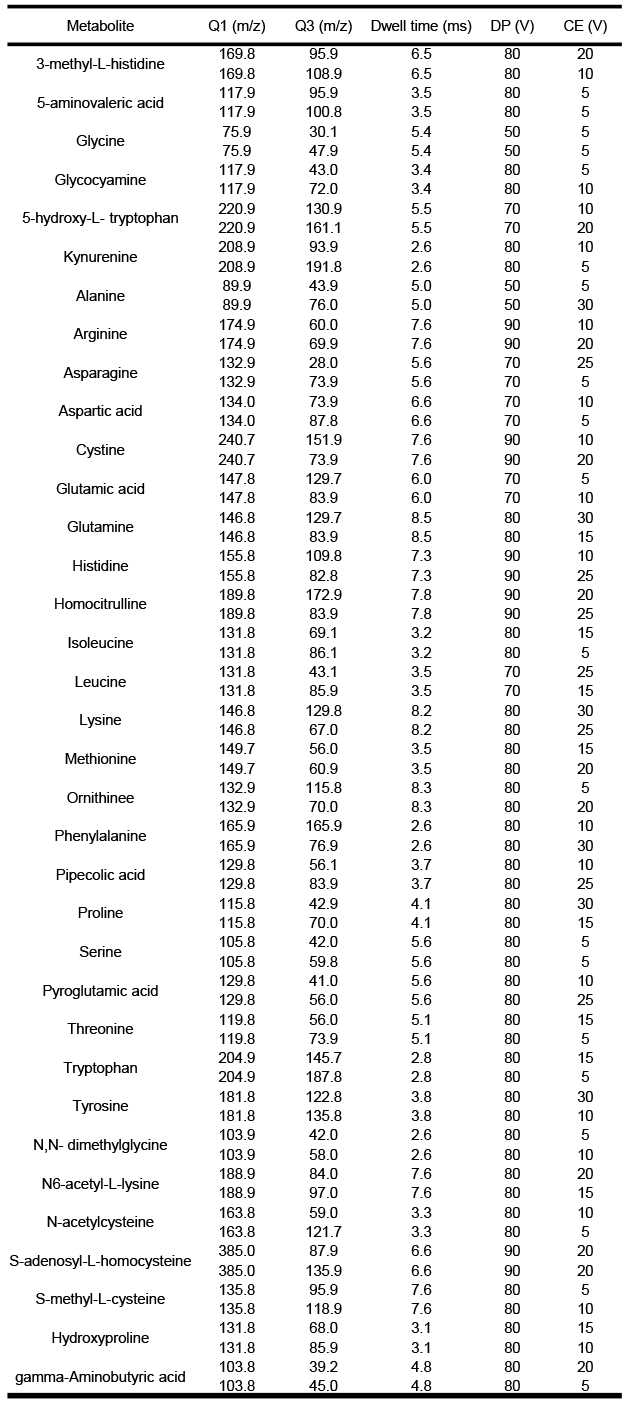


**Supplementary Table 1.** Optimization of MS parameters of target amino acids. DP delustering potential, CE collision of energy.

**Supplementary Table 2.** List of packages used for the analysis and optimal parameters for model.

**
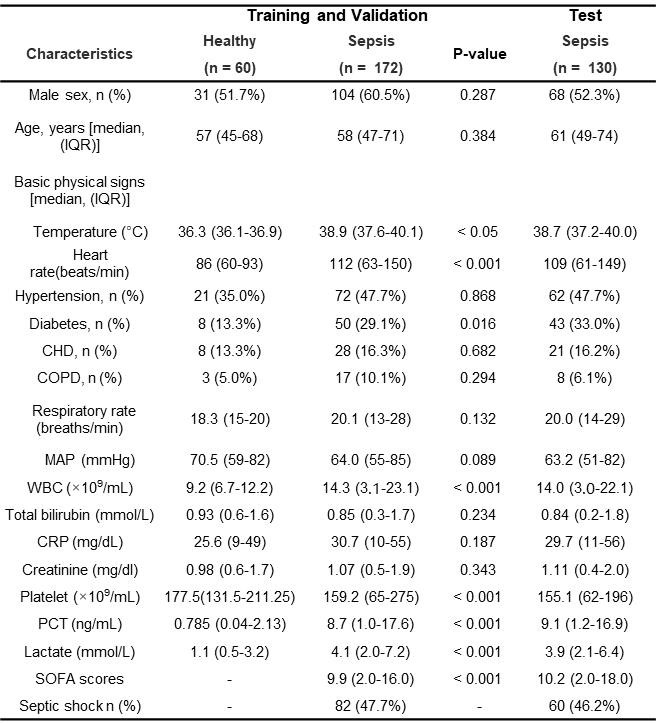
**

**Supplementary Table 3.** Detailed Demographic and clinical characteristics of the enrolled participants.

**
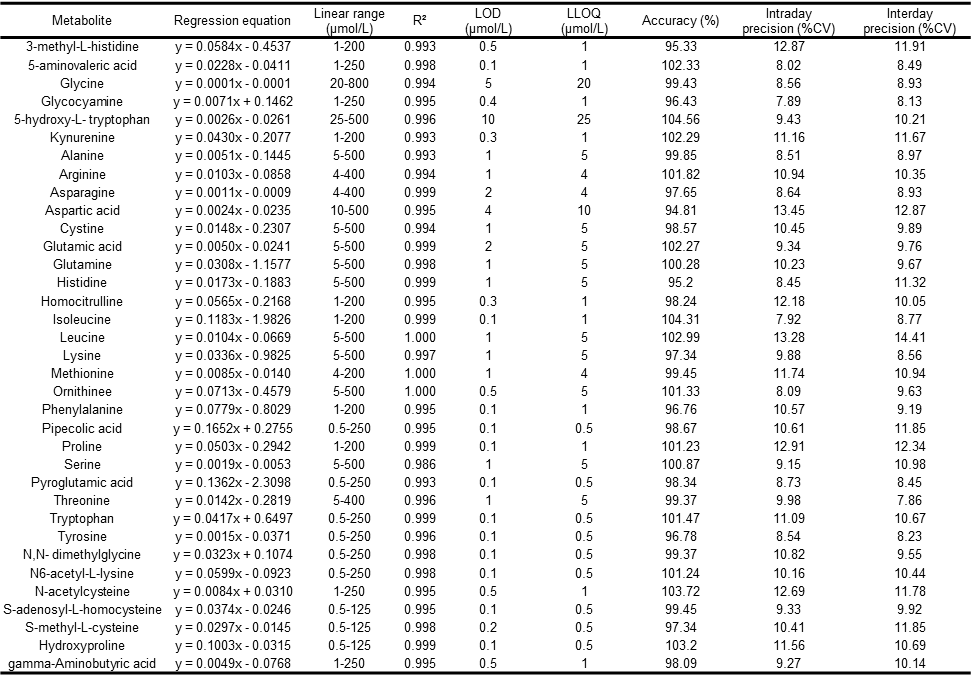
**

**Supplementary Table 4.** Analytical performance parameters of the UPLC–MS/MS-based quantitative platform for amino acid analysis. The evaluated parameters include the linear range, limit of detection (LOD), lower limit of quantification (LLOQ), accuracy, and precision. Quality control (QC) samples at different concentration levels were used to assess intra- and inter-day precision, expressed as coefficient of variation (CV).

**
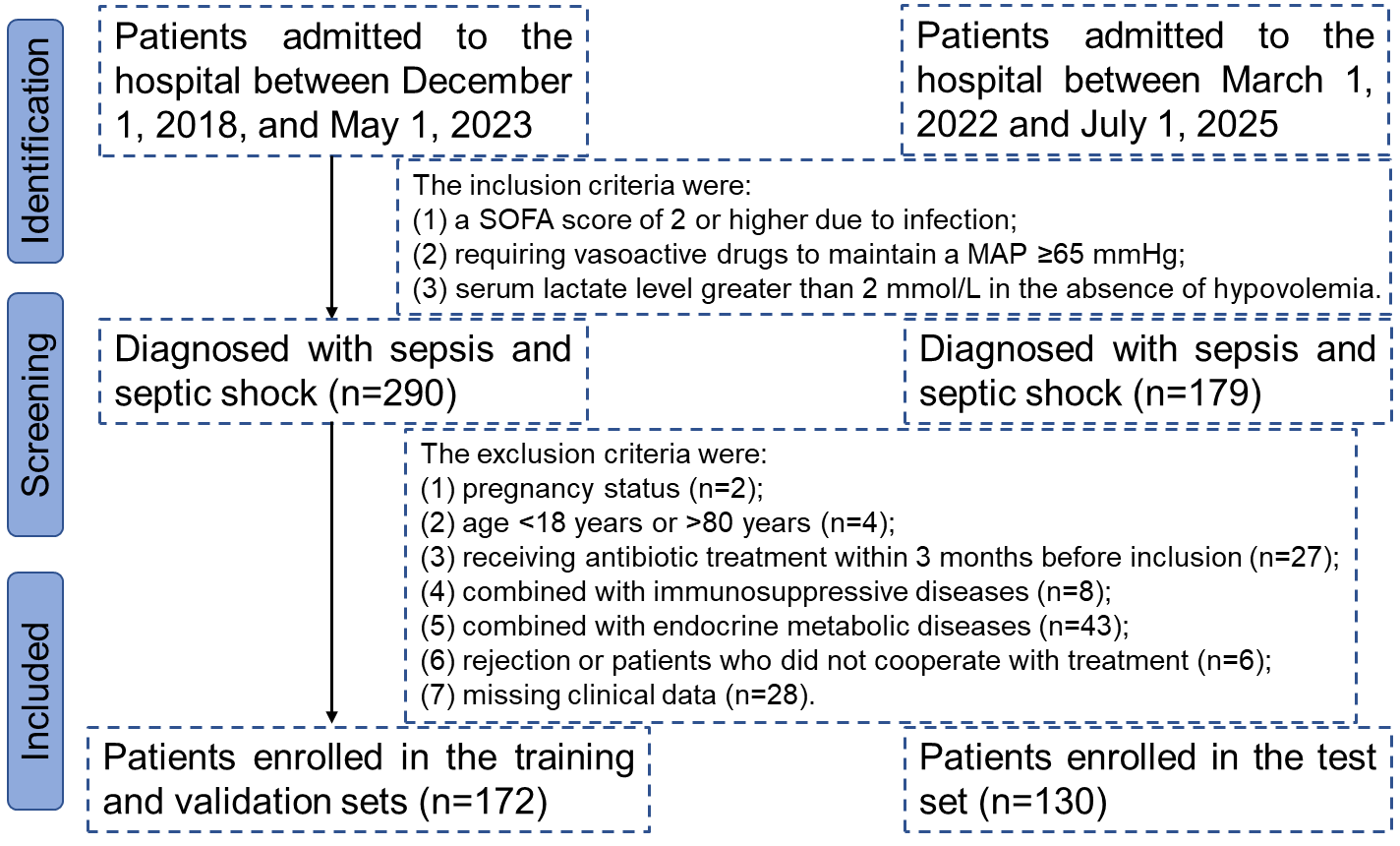
**

**Figure S1.** Flowchart of the inclusion and exclusion process of patients in this study.


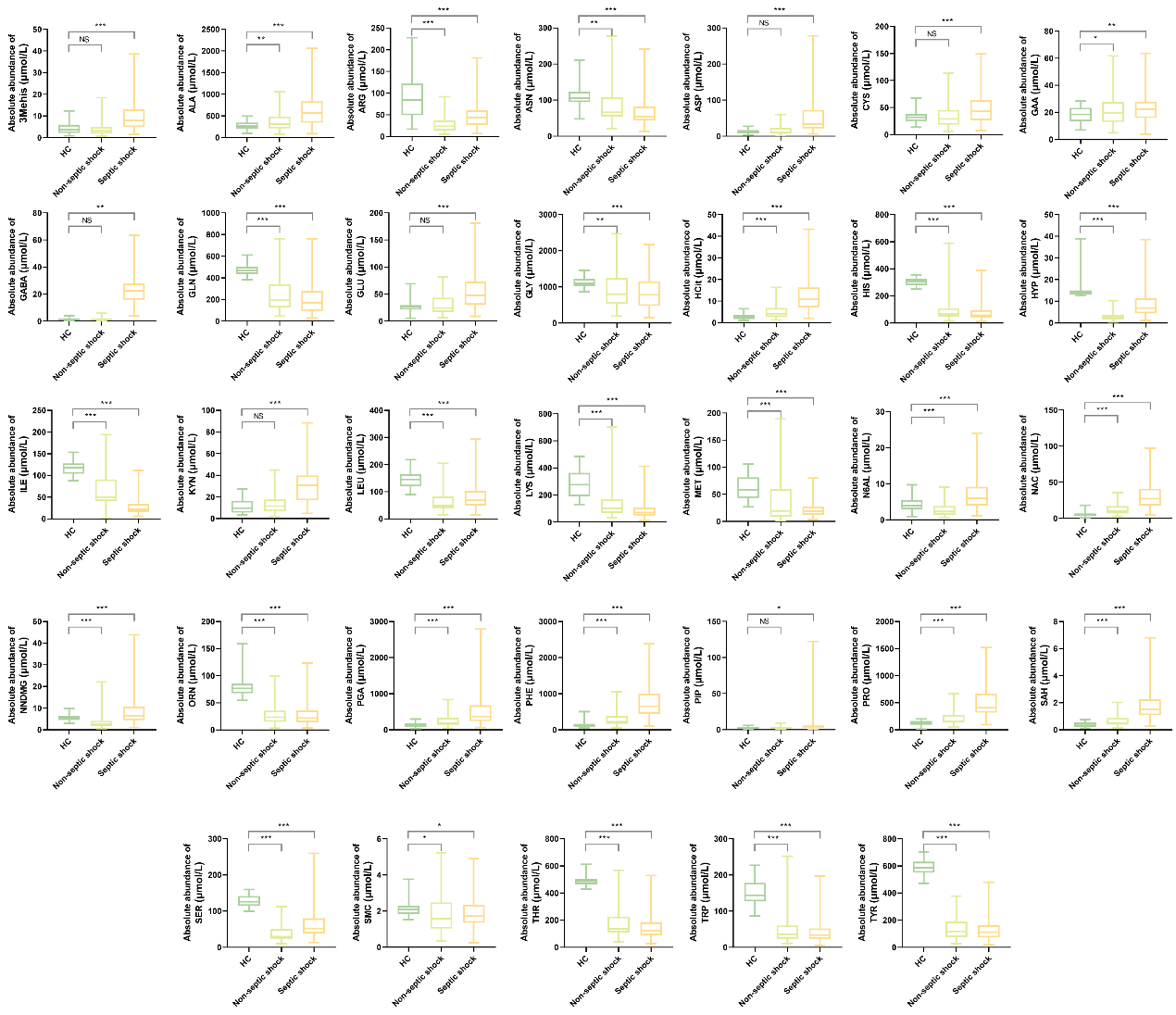


**Figure S2.** Abundance of amino acid profiles in healthy control (HC) group, patients with sepsis and patients with septic shock.

**
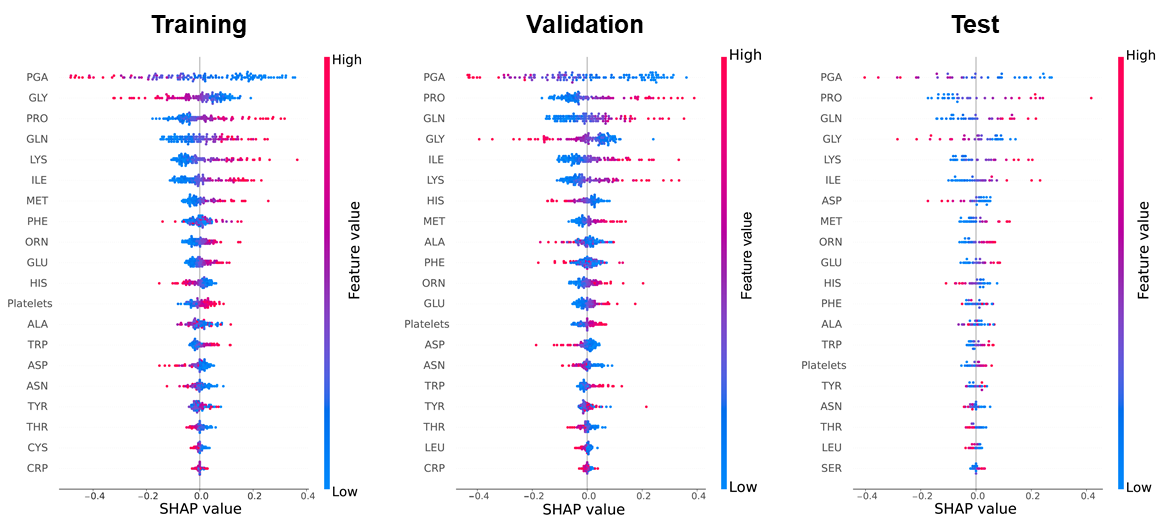
**

**Figure S3.** SHapley Additive exPlanation (SHAP) values of the top 20 features of the Deephit model.


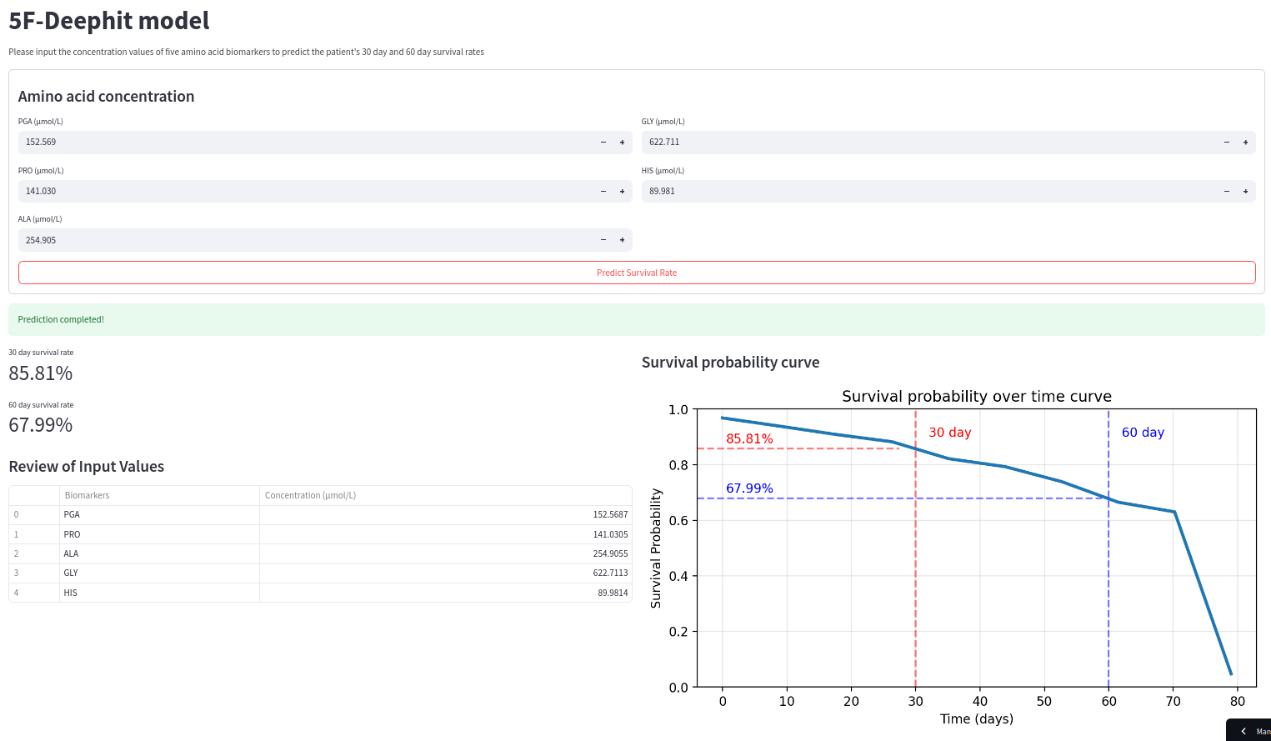


**Figure S4.** An online web application according to the final Deephit model with five features.

**
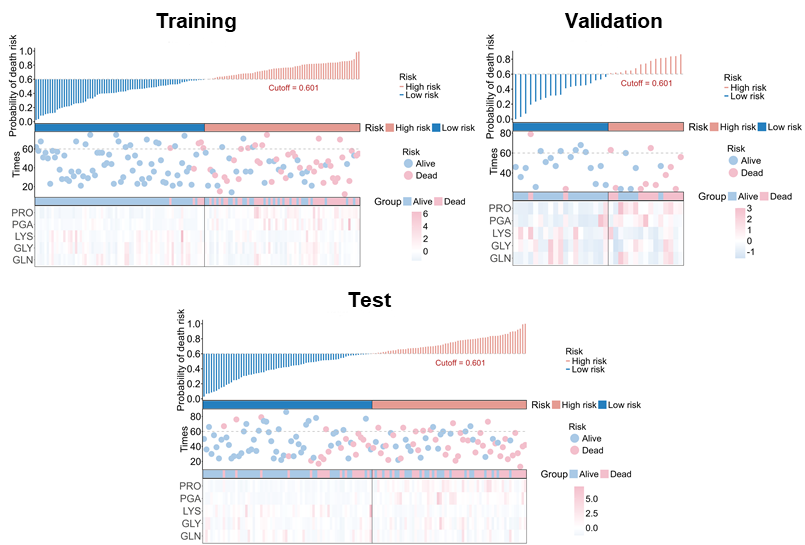
Figure S5.** Construction of the symptom formation 60-day probability of death risk for each patient in the training and independent set based on the 5F-Deephit model.


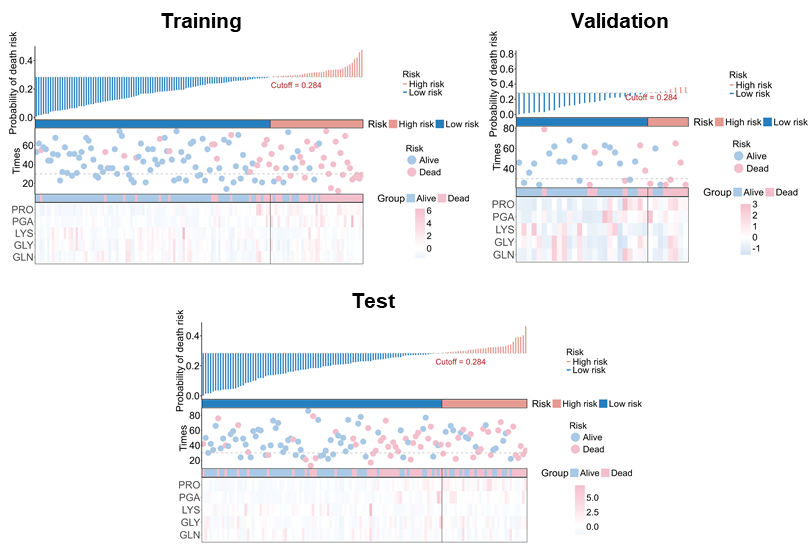


**Figure S6.** Construction of the symptom formation 30-day probability of death risk for each patient in the training and independent set based on the 5F-Deephit model.

**
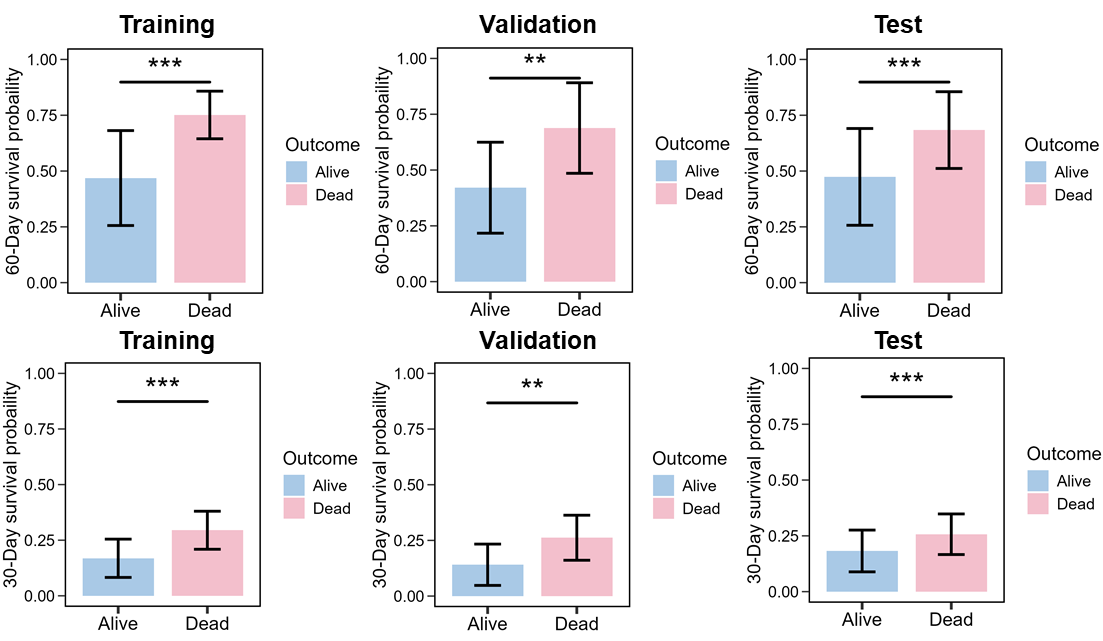
**

**Figure S7.** Statistical analysis of death risk probability between different clinical prognosis in both the training and test sets.

**
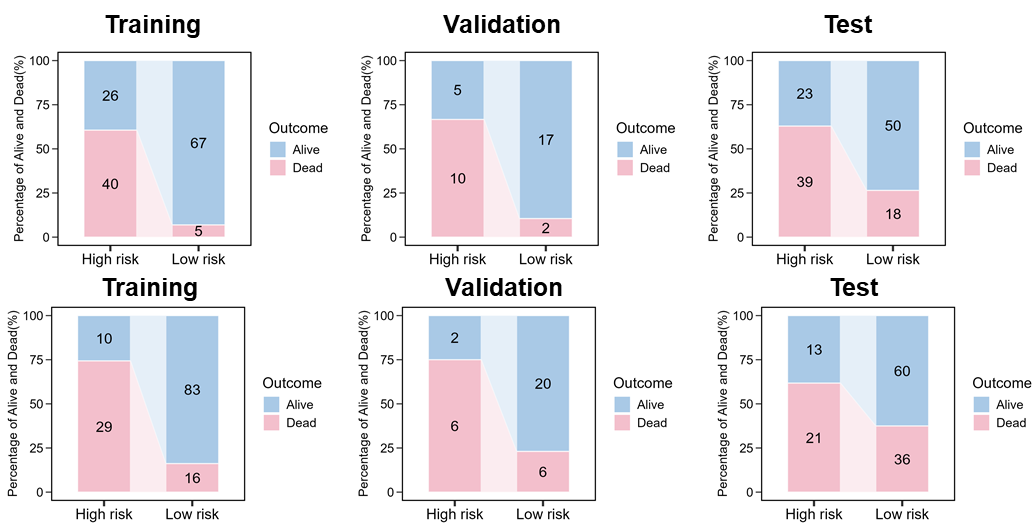
**

**Figure S8.** Statistical analysis of percentage of different clinical prognosis (60-day prognosis in the top row and 30-day prognosis in the bottom row) between high and low-risk group.
